# Alix is required during development for normal growth of the mouse brain

**DOI:** 10.1038/srep44767

**Published:** 2017-03-21

**Authors:** Marine H. Laporte, Christine Chatellard, Victoria Vauchez, Fiona J. Hemming, Jean-Christophe Deloulme, Frédérique Vossier, Béatrice Blot, Sandrine Fraboulet, Rémy Sadoul

**Affiliations:** 1 Institut National de la Santé et de la Recherche Médicale (INSERM), U1216, F-38042 Grenoble, France; 2 Université Grenoble Alpes, Institut des Neurosciences, F-38042 Grenoble, France

## Abstract

Alix (ALG-2 interacting protein X) drives deformation and fission of endosomal and cell surface membranes and thereby intervenes in diverse biological processes including cell proliferation and apoptosis. Using embryonic fibroblasts of Alix knock-out mice, we recently demonstrated that Alix is required for clathrin-independent endocytosis. Here we show that mice lacking Alix suffer from severe reduction in the volume of the brain which affects equally all regions examined. The cerebral cortex of adult animals shows normal layering but is reduced in both medio-lateral length and thickness. Alix controls brain size by regulating its expansion during two distinct developmental stages. Indeed, embryonic surface expansion of the Alix ko cortex is reduced because of the loss of neural progenitors during a transient phase of apoptosis occurring between E11.5 and E12.5. Subsequent development of the Alix ko cortex occurs normally until birth, when Alix is again required for the post-natal radial expansion of the cortex through its capacity to allow proper neurite outgrowth. The need of Alix for both survival of neural progenitor cells and neurite outgrowth is correlated with its role in clathrin-independent endocytosis in neural progenitors and at growth cones. Thus Alix-dependent, clathrin independent endocytosis is essential for controlling brain size.

The size of adult brains is the resultant of a delicate balance between neural progenitor proliferation, differentiation and neurite outgrowth, which occur during both embryogenesis and post-natally. A multitude of cell surface receptors and their downstream signaling harmoniously orchestrate these processes to allow proper brain development. The endolysosomal system is crucial for this orchestration, not only by regulating cell surface expression and degradation of the receptors, but also by organising signaling hubs inside endosomes.

Alg-2 interacting protein X (Alix/PDCD6IP) is a cytosolic protein^1^ acting at the plasma membrane to allow clathrin-independent endocytosis (CIE) of receptors through its interaction with endophilins^2^,^3^ and at endosomes to regulate caspase activation through binding to proteins of the endosomal sorting complexes required for transport (ESCRT)^4^,^5^. Alix is ubiquitously expressed^1^ and is also involved in virus egress, cytokinesis, cell spreading and membrane repair^6^ which all rely on ESCRT-mediated membrane deformation and fission. In order to better appreciate the role of the protein *in vivo*, we have made an Alix knock out (ko) mouse line and report here that the mice are viable and appear normal except for severe reduction in size of testes and brain as well as hydrocephaly. We focused our present study of the Alix ko mice on deciphering the mechanisms leading to microcephaly.

During early development, proliferation of neuroepithelial cells surrounding the ventricle allows expansion of the telencephalon giving rise to most of the brain including the cerebral cortex^7^. Neuroepithelial cells mature into radial glial cells (RGCs), which continue to proliferate. At the onset of neurogenesis around E11.5, RGCs start to give birth to deep-layer neurons or intermediate progenitors, which further divide to produce more neurons^8^,^9^. Newly generated neurons migrate radially along RGC processes towards the pial surface and settle to build the cortical neuron layers^10^,^11^. Subsequently, neurons elaborate axon and dendrites, the growth of which accounts for most of the post-natal lateral expansion of the cortex^12^.

Here, we demonstrate that one aspect of the microcephaly observed in Alix ko mice is partly due to a massive but transient wave of apoptosis of a fraction of neural progenitors in the E11.5 and E12.5 telencephalon correlating with a reduced lateral expansion of the tissue and loss of deep-layer neurons. Subsequent developmental steps appear normal giving rise to newborn (P0) cortices with reduced volumes but normal cortical layers except for thinner layer VI. However, post-natal radial expansion is altered giving rise to an adult cortex with all layers significantly thinner. This alteration correlates with a reduction in post-natal neurite extension detected both *in vivo* and *in vitro*. Thus, Alix plays a major role in determining the size of the brain by controlling neural progenitor survival at the start of neurogenesis and later on, by regulating post-natal dendrite development.

## Results

### Alix ko mice suffer from severe microcephaly

**Figure 1:**
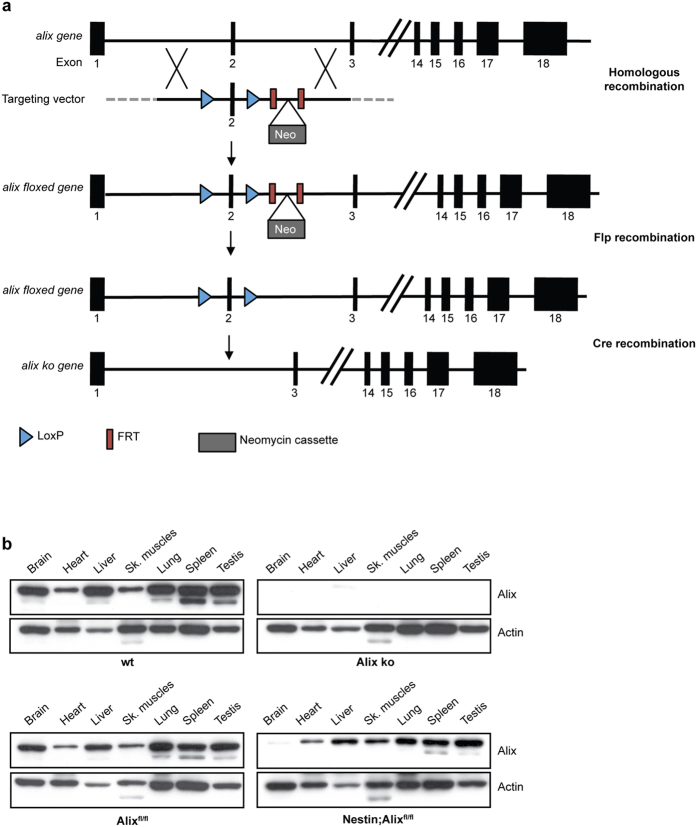
Strategy used for making Alix ko mice. (**a**) Constructs used for the recombination of *alix*. Alix-floxed mice were crossed with Actin-cre mice or Nestin-cre mice. (**b**) Cropped images of Western blot demonstrate the lack of Alix expression in all organs of Alix ko mice (upper panel) and a strong reduction in the brain of Nestin;Alix^fl/fl^ mice (lower panel).

*Alix* floxed animals were crossed with mice expressing Cre under an actin promoter to knock out *alix* ubiquitously (Fig. 1a,b, see Supplementary Fig. S7 for full-length Western blot). Homozygous Alix ko animals were viable even though there was a significant reduction in the proportion of Alix ko born from crossing Alix heterozygotes (15% vs the expected 25% Mendelian ratio), thus the lack of *alix* leads to a lethal phenotype with varying penetrance. Mendelian ratios were, however, normal in E12.5 litters demonstrating that lethality must occur beyond this developmental stage (Supplementary Fig. S1a).

**Figure 2:**
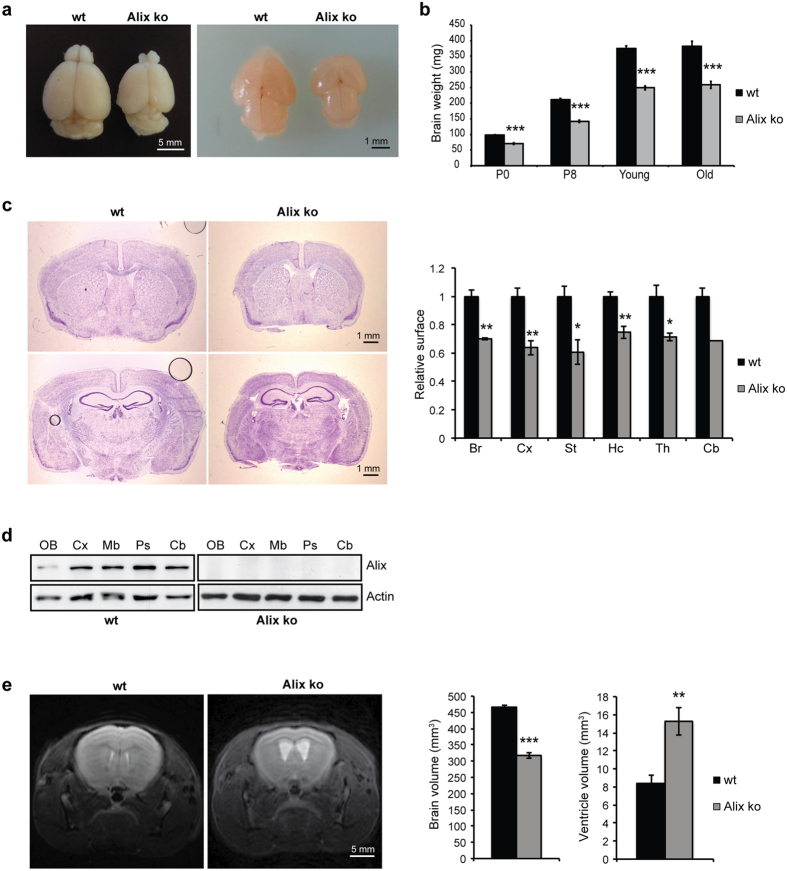
Microcephaly of Alix ko mice. (**a**) Whole brain of adult (left panel) and newborn mice (right panel) of the indicated genotype. (**b**) Whole brain weight from P0, P8, young (2 months) and old mice (>10 months) (n = 6 animals per age ***p < 0.001). (**c**) Coronal sections of adult brain stained with Cresyl violet used for quantification of surface areas of brain (Br), cortex (Cx), striatum (St), hippocampus (Hc), thalamus (Th) and cerebellum (Cb) (n = 4 animals *p < 0.05; **p < 0.01). (**d**) Cropped images of Western blot revealing Alix expression in olfactory bulb (OB), cortex (Cx), midbrain (Mb), pons (Ps) and cerebellum (Cb) from adult mice shows ubiquitous Alix expression in wt, and absence from Alix ko brains. (**e**) MRI sections of adult brains show volume reduction and increase in ventricle size (n = 5 animals **p < 0.01; ***p < 0.001).

Alix ko mice had a normal life span and the body weight of embryos, pups and adults was not different between ko and wild type (wt) littermates (Supplementary Fig. S1b). The size of adult animals was equivalent in wt and Alix ko animals (Supplementary Fig. S1c). No gross organ abnormalities were detected, except for testes which were severely reduced in size, but had an apparently normal organization (Supplementary Fig. S1d,e). The only other obvious difference in the ko was the significant reduction in their brain size (Fig. 2), already obvious at P0 (Fig. 2a) and reflected by a decreased brain weight at all ages studied (Fig. 2b). Coronal sections of 3-month old adult brains, showed no gross abnormalities in overall brain architecture despite a 20 to 30% decrease in size of all regions examined (Fig. 2c) in agreement with Alix expression in these regions (Fig. 2d, see Supplementary Fig. S8 for full-length Western blot). Magnetic resonance imaging (MRI) sections of live adults confirmed a 30% reduction in total brain volume and revealed hydrocephaly with ventricles almost doubled in size (Fig. 2e).

### Alix ko cortex is thinner but normally layered

**Figure 3:**
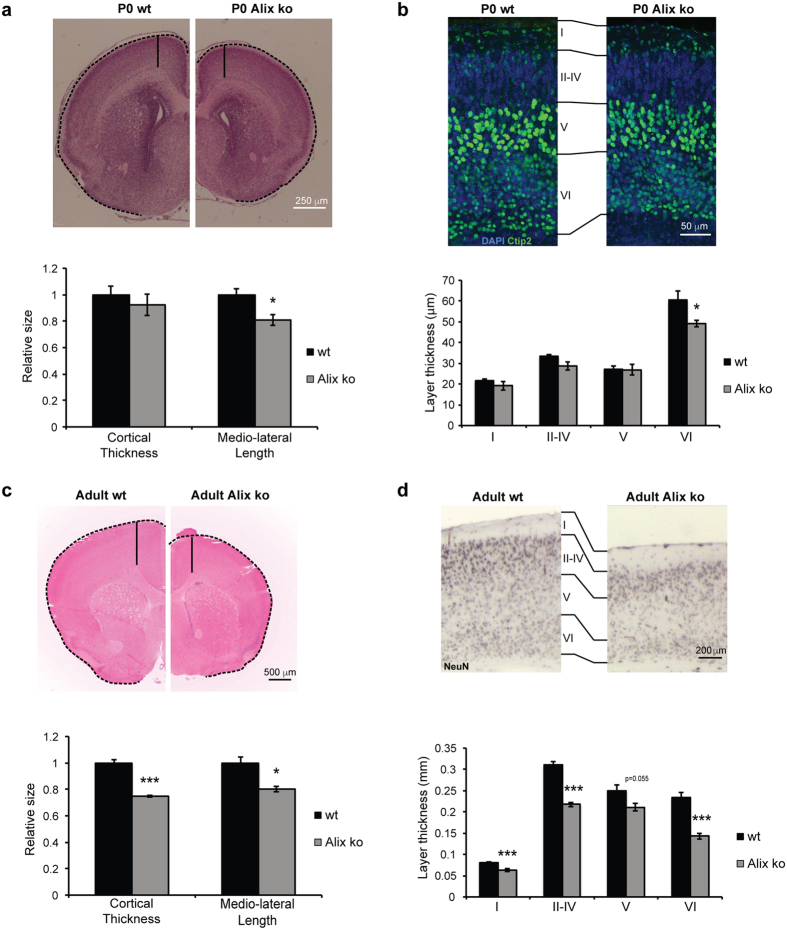
Reduction in size and thickness of Alix ko cortices. (**a**,**c**) Coronal sections of P0 (**a**) and adult (**c**) brains stained with haematoxylin/eosin. Quantifications show that the cortical medio-lateral length (dashed lines) is reduced in P0 and the adult, while the radial thickness (solid lines) is reduced in the adult only (n = 4 animals *p < 0.05; ***p < 0.001). (**b**,**d**) Sections of P0 (**b**) and adult (**d**) motor cortex labeled with anti-Ctip2, (**b**) and anti-NeuN (**d**) antibodies to discriminate cortical layers (LI-LV/VI). Measurements reveal thinning of all layers in adult (**d**), but only of layer VI in P0 Alix ko brains (**b**) (n = 7 animals for P0, n = 4 animals for adults *p < 0.05; **p < 0.01; ***p < 0.001).

We then focused our study on the neocortex, hereafter referred to as cortex. Comparison of sections of Alix ko vs wt P0 cortices demonstrated a 20% reduction in the outer medio-lateral length only (Fig. 3a, dashed line), whereas the radial dimension (solid line) was not significantly reduced. Both the thickness and layering of the P0 cortex were apparently normal, apart from layer VI revealed by Ctip2^13^ that was 20% thinner (Fig. 3b). Thus, Alix is necessary during early embryogenesis for the lateral expansion of the cortex. This requirement must be early and transient enough not to disturb the overall layering of the cortex except for the deepest layer VI made of the first-born neurons. In contrast to P0, the cortex of adult Alix ko animals was reduced in both medio-lateral length (25%) and thickness (20%) (Fig. 3c). Reduction in thickness was detected in all layers with the most striking difference seen in layer VI, which was decreased by 40% (Fig. 3d). Thus Alix is essential for two steps of corticogenesis: the embryonic lateral expansion of the telencephalon and the post-natal thickening of cortical layers.

### The ventricular surface and number of progenitors are reduced in Alix ko embryos

**Figure 4:**
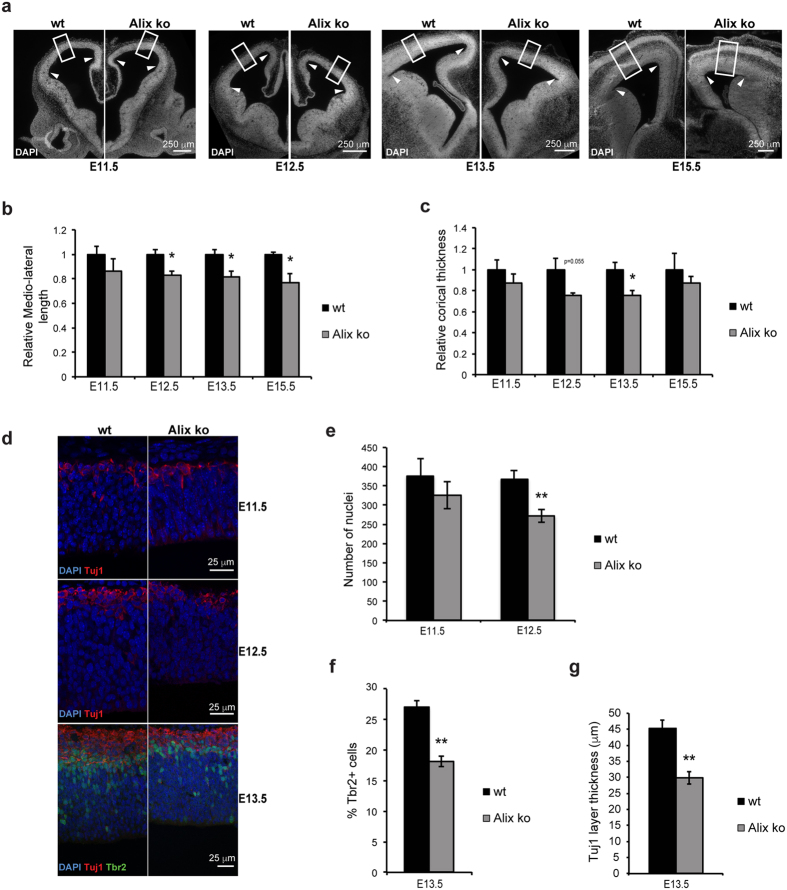
Reduced cortical size in Alix ko embryos correlates with a reduced number of intermediate progenitors and neurons. (**a**) DAPI stained coronal sections of E11.5, E12.5, E13.5, E15.5 embryonic brains. Arrowheads and white frames in (**a**) show examples of regions used for measurements of the medio-lateral length at the ventricule side of the dorsal telencephalon (**b**) and the cortical thickness (**c**) (n = 3 embryos per stage *p < 0.05). (**d**) Single plane confocal image of dorsal telencephalon labeled with DAPI (blue) and anti-Tuj1 (red) (E11.5, E12.5), and Tbr2 (green) (E13.5). (**e**,**f**) Reduction in the number of nuclei at E12.5 (**e**) and in the percentage of Tbr2 + progenitors at E13.5 (**f**). Counts were performed in a rectangle with a 150 μm base along the ventricle and the height of the cortical wall (n = 3 embryos per stage **p < 0.01). (**g**) The Tuj1 + neuron layer is thinner in Alix ko E13.5 cortices (n = 3 embryos **p < 0.01).

The sole reduction in the medio-lateral length of P0 cortices suggests that *alix* ablation affects the number of neural progenitor cells (NPCs). We therefore studied early embryogenesis in the dorsal telencephalon which gives rise to the cortex. We found that, in Alix ko embryos, the medio-lateral length of the dorsal telencephalon at the ventricular side (Fig. 4a, arrowheads and Fig. 4b) as well as the radial thickness (Fig. 4a, frame and Fig. 4c) were reduced at E12.5 and E13.5. In E12.5 Alix ko telencephalon, the reduced radial thickness correlated with a 30% drop in the number of DAPI stained nuclei which mostly correspond to RGCs (Fig. 4d,e). Consistent with a reduced number of RGCs, the number of Tbr2-positive (Tbr2+) intermediate progenitors, directly generated from these cells, was decreased by 30% in Alix ko E13.5 telencephalon (Fig. 4f). Furthermore, a 35% reduction in the thickness of the Tuj1 + newly-formed neuronal layer was observed in E13.5 Alix ko brains (Fig. 4g, supplementary Fig. S2). This strongly suggests that the Alix-lacking telencephalon contains fewer RGCs and, consequently, fewer Tbr2 + intermediate progenitors and Tuj1 + neurons, which differentiate from these progenitors.

### The lack of Alix expression results in transient apoptosis of neural progenitors

**Figure 5:**
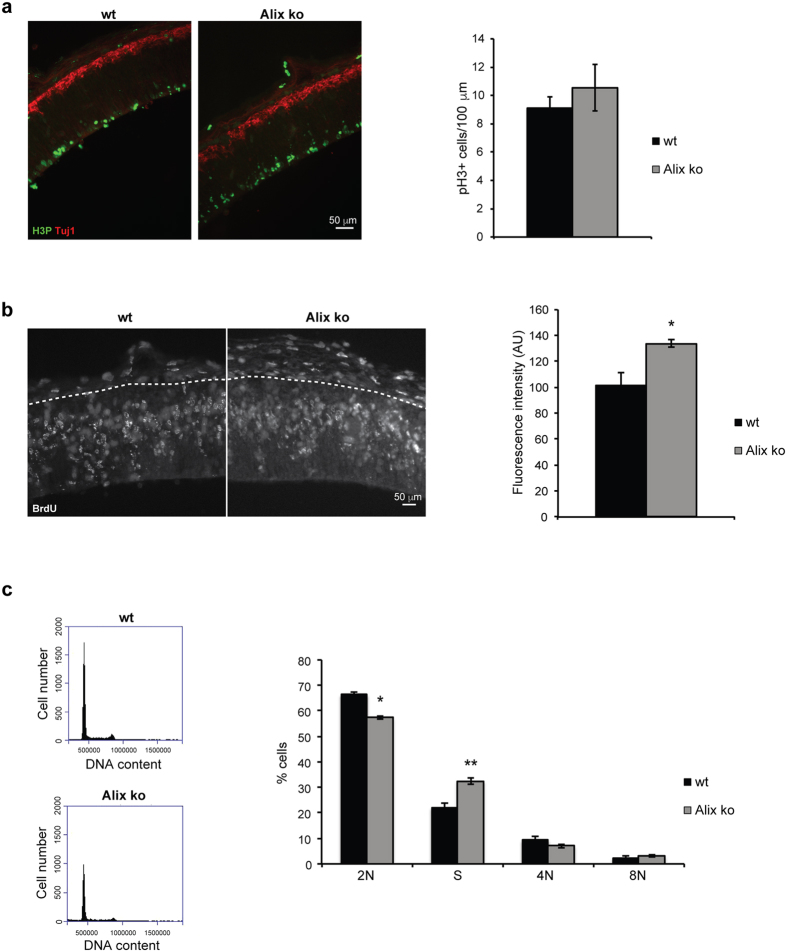
Modified cell proliferation does not account for the reduced cortical size of Alix ko brains. (**a**) E12.5 cortices stained with anti-phospho-histone H3 to label M-phase nuclei (pH3, green), and anti-Tuj1 to label neurons (red). Cells were counted in a rectangle with a 100 μm base along the ventricle and the height of the cortical wall (n = 3 embryos). (**b**) BrdU incorporation is increased in Alix ko cortex. Sections were stained with anti-BrdU antibody. Dashed lines delimit the basal surface of the cortex. Bar chart shows the relative fluorescence intensities (n = 3 embryos *p < 0.05). (**c**) Flow cytometry of cells freshly dissociated from E12.5 cortices. Left panels show profiles of DNA content of wt and ko cells stained with propidium iodide. The average percentage of cells in each cell cycle phase shows a decrease in G1/ GO (2 N), an increase in S-phase, but no increase in polyploid (4 N and 8 N) cells in Alix ko cortices (n = 3 embryos *p < 0.05; **p < 0.01).

The reduced number of neural progenitors in Alix ko animals could result from a decrease in cell proliferation. However, the number of cells undergoing mitosis revealed by phospho-Histone H3 expression was not significantly different between Alix ko and wt E12.5 cortices (Fig. 5a). Remarkably, bromodeoxyuridine (BrdU) incorporation in the ventricular zone (VZ) was higher in Alix ko embryos (Fig. 5b) and flow cytometry analysis of dissociated E12.5 cortical cells confirmed an increased number of cells in S-phase and a decrease of those in G1/G0 (denominated 2 N in Fig. 5c) in the ko cortices. We could not detect any increase in 8 N cells, indicating possible polyploidy, suggesting that in NPCs the lack of Alix does not compromise completion of mitosis (Fig. 5c). Thus, the lack of Alix seems to increase the length of the S-Phase without affecting the number of cells undergoing mitosis, suggesting that the downsizing of Alix ko brains does not result from a reduced proliferation of NPCs.

**Figure 6:**
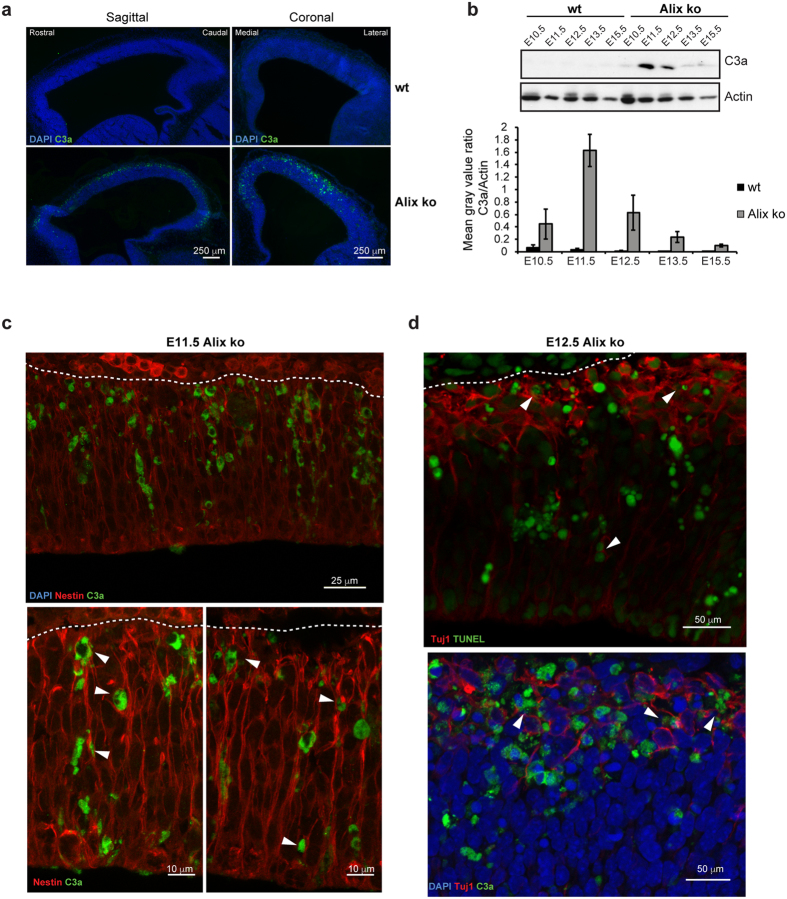
Massive apoptosis in Alix ko cortices during early development. (**a**) Sections of E12.5 forebrains labeled with anti-active caspase-3 (C3a, green) show dying cells scattered along the entire antero-posterior and medio-lateral axes of Alix ko cortices. (**b**) Cropped images of Western blot of extracts from dorsal telencephalons shows that C3a is only detectable at E11.5 and E12.5 Alix ko cortices. Bottom panel shows quantifications (n = 4 embryos per stage). (**c**) Maximal projection confocal images of double C3a (green) and RC2 (red) staining of Alix ko E11.5 cortex. Upper photograph shows apical to basal alignment of C3a + cells. Higher magnification (lower panels) shows RC2 + RGCs filled with C3a (arrowhead). Dashed lines delimit the basal surface of the cortex. (**d**) Double staining of E12.5 coronal sections for Tuj1 (red) and TUNEL (green, upper panel) or C3a (green, lower panel), show dying cells spread throughout the cortical wall. Arrowheads indicate colocalization between Tuj1 and TUNEL or C3a. Dashed lines delimit the basal surface of the cortex.

Another possible explanation for the observed reduction in the cortex size is an increase in cell death at early embryonic stages. Indeed, many cells expressing the active form of caspase-3 (C3a) were observed throughout the entire telencephalon of E12.5 Alix ko embryos (Fig. 6a lower panels) in contrast to the absence of C3a staining in wt embryos (Fig. 6a upper panels). Western blot analysis of dorsal telencephalon revealed a sudden rise of caspase-3 activation peaking at E.11.5, and declining at E12.5 to become almost undetectable at E13.5 (Fig. 6b, see Supplementary Fig. S9 for full-length Western blot). On E11.5 telencephalon sections, C3a labeled cells were often clustered and aligned radially (Fig. 6c). Some of these cells expressed Nestin (revealed by an anti-RC2 antibody), a marker of RGCs (arrowheads Fig. 6c, lower panel). In contrast, at E12.5, TUNEL and C3a + cells were more scattered and abundant in the basal half of the telencephalon (Fig. 6d). Some dying cells within the preplate were Tuj1 + newly-born neurons (arrowheads, Fig. 6d). Thus, our results suggest that Alix is required for the survival of some RGCs and of their progeny at the onset of neurogenesis. Of note is that dying cells are sporadic in Alix ko, whereas Alix seems equally expressed by all cells of the E12.5 wt telencephalon (Supplementary Fig. S3). Another striking feature of E11.5-13.5 Alix ko telencephalon is the presence of sparse aggregates of pyknotic nuclei throughout the cortical wall (Supplementary Fig. S4a, arrowhead). Pyknotic nuclei-containing aggregates were not seen at E15.5 (data not shown), an age when C3a could no longer be detected (Fig. 6b). The aggregated pyknotic nuclei were surrounded by IBA1 + microglia and thus appear to represent phagocytosed dead cells (Supplementary Fig. S4b). This was further suggested by electron microscopy observation of apoptotic body clusters surrounded by a healthy engulfing cell (Supplementary Fig. S4c). Thus, apoptosis occurring within the Alix ko telencephalon is accompanied by increased microglial activation allowing the disposal of apoptotic bodies.

In conclusion the lack of Alix expression induces a transient wave of apoptosis of neural progenitors at the beginning of neurogenesis, which compromises lateral expansion and integrity of layer VI neurons. This abnormal developmental phase is transient enough to give rise to almost normal cortex layering with reduced layer VI width at P0.

### Signal transduction is deregulated in Alix ko NPCs

**Figure 7:**
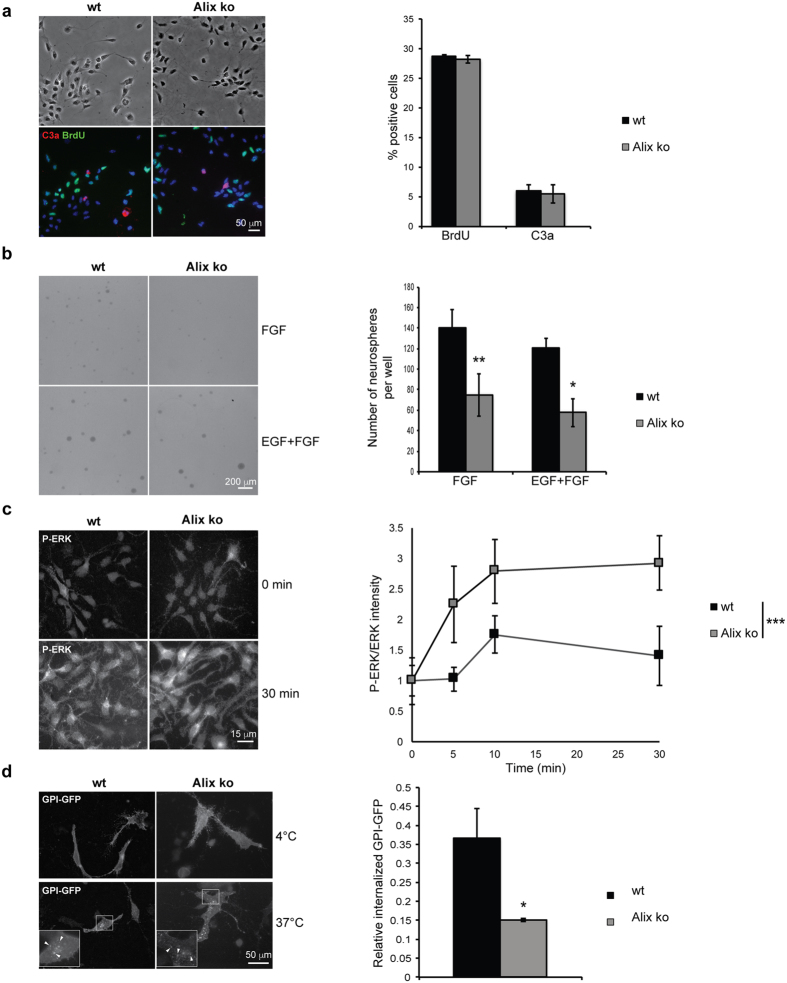
Alix ko NPCs in culture do not undergo abnormal apoptosis but are deficient in endocytosis and FGF signaling. (**a**) NPCs were dissociated from E12.5 embryos, cultured for 3 DIV and apoptotic cells immunostained for C3a and BrdU revealing no difference in proliferation or apoposis (n = 3 embryos). (**b**) Alix ko NPCs form fewer neurospheres in culture. Cells dissociated from E12.5 embryos were grown for 6 days on a non permissive substrate in presence of FGF or EGF and FGF. Graphs show the number of neurospheres formed from 1000 seeded cells prepared from embryos of each genotype (n = 6 for wt and n = 5 for Alix ko *p < 0.05; **p < 0.01). (**c**) FGF signaling is impaired in Alix ko NPCs. NPCs were treated with FGF-2 for the indicated times and double immunostained for ERK and phospho-ERK. Mean fluorescence values per cell were estimated and the ratio of P-ERK/ERK normalized to t = 0 (n = 4 embryos ***p < 0.001). (**d**) Clathrin-independent endocytosis is severely impaired in cultured NPCs. Endocytosis was assessed on 6DIV GPI-GFP expressing NPCs. Cells were first incubated at 4 °C with antibodies against GFP, and endocytosis triggered by incubation at 37 °C. Representative photographs of wt and Alix ko NPCs in which endocytosis was triggered (arrowheads indicate GPI-GFP containing endocytic vesicles). Quantification shows the strong reduction in GPI-GFP internalized in Alix ko NPCs (n = 4 embryos *p < 0.05).

In order to elucidate the molecular basis of the cell loss seen *in vivo*, we used cultures of dissociated NPCs from E12.5 telencephalon. The levels of apoptosis and proliferation in presence of Fibroblast Growth Factor (FGF) and Epidermal Growth Factor (EGF) were the same in wt and ko cells cultured for 3 days on laminin (Fig. 7a). However, when NPCs were cultured on non permissive substrates to form neurospheres, the number of neurospheres was strikingly reduced in Alix ko cultures (Fig. 7b). Since FGF signaling is required to maintain the self-renewal of neurosphere forming cells^14^ we next tested whether ERK1/2 phosphorylation triggered by FGF-2 was affected by the absence of Alix expression. We found that this is indeed the case since ERK1/2 phosphorylation induced by FGF-2 was abnormally high in Alix ko cells suggesting a misregulation of the receptor (Fig. 7c). Using MEF cells we have recently shown that Alix is required for clathrin-independent endocytosis (CIE) of several surface receptors and thereby regulates signaling^3^. To test the state of CIE in NPCs, we studied the fate of glycosyl-phosphatidyl-inositol (GPI) anchored proteins which are specifically endocytosed by CIE. Using NPCs expressing GPI-GFP, we found that internalization of the protein was reduced by more than 50% in Alix ko cells compared to wt cells (Fig. 7d). This defect might explain the abnormal FGF signalization, known to be regulated by CIE^15^,^16^ and could be related to our developmental microcephaly phenotype since FGF is known to control NPC fate during early development^17^,^18^,^19^.

### Lack of Alix expression impairs post-natal dendrite development

Our observations strongly suggest that during cortical development the lack of Alix disrupts two distinct processes that are temporarily dissociated: one during early embryogenesis regulates lateral expansion and survival of deep layer neurons, while the other allows post-natal radial expansion. To verify our hypothesis, we used conditional Alix ko animals (Nestin;Alix^fl/fl^) obtained from crossing Alix-floxed mice (Alix^fl/fl^) with Nestin-cre mice (Fig. 1b, Supplementary Fig. S5a). Indeed, Liang *et al*. demonstrated that in Nestin-cre embryos, recombination begins in cells of the cortical plate and in sparse cells of the VZ only at E12.5^20^, a stage at which apoptosis already declines in Alix ko embryos. Accordingly, we found that in contrast to Alix ko and similarly to wt animals, Alix is still expressed and no apoptotic cells could be detected in E12.5 Nestin;Alix^fl/fl^ cortices (Supplementary Fig. S5b,c). In adult mice, the reduction in the medio-lateral length of the cortex was far less pronounced in Nestin;Alix^fl/fl^ mice than in Alix ko mice (8% vs 20% respectively), while reduction in the cortical thickness was similar in both lines (Supplementary Fig. S5d). These observations directly demonstrate that besides controlling cell death of progenitors and thereby lateral expansion of the embryonic cortex, Alix is also necessary for the radial growth of cortical layers.

**Figure 8:**
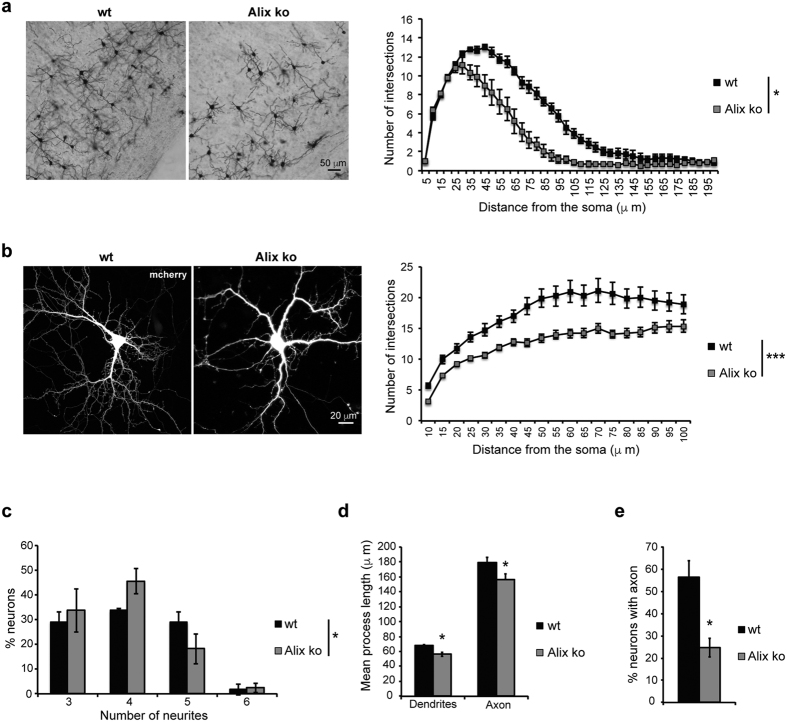
Dendritic arborisation is reduced in Alix ko neurons both *in vivo* and *in vitro.* (**a**) Golgi-stained cortical neurons in coronal brain sections of 2-month-old mice. Sholl analysis of dendrites from neurons in layers V-VI. Mean numbers of intersections between dendrites and concentric circles are shown as a function of the distance (μm) of the circles from the soma (n = 3 animals *p < 0.05). (**b**) Confocal images of wt or Alix ko, 13 DIV hippocampal neurons expressing mCherry. Sholl analysis indicates the reduction in dendrite arborisation of Alix ko neurons (n = 70 neurons ***p < 0.001). (**c**,**d**,**e**) 1DIV hippocampal neurons stained with anti-MAP2 and anti-Tau to discriminate between dendrites and axon were used for the following quantifications: (**c**) Percentage of neurons with 3, 4, 5 or 6 neurites showing that neurons lacking Alix have fewer neurites, (**d**) reduced length of dendrites and axon growing from Alix ko neurons, (**e**) percentage of neurons with an axon is strongly decreased in Alix ko neurons (n = 3 independent experiments *p < 0.05; **p < 0.01).

**Figure 9:**
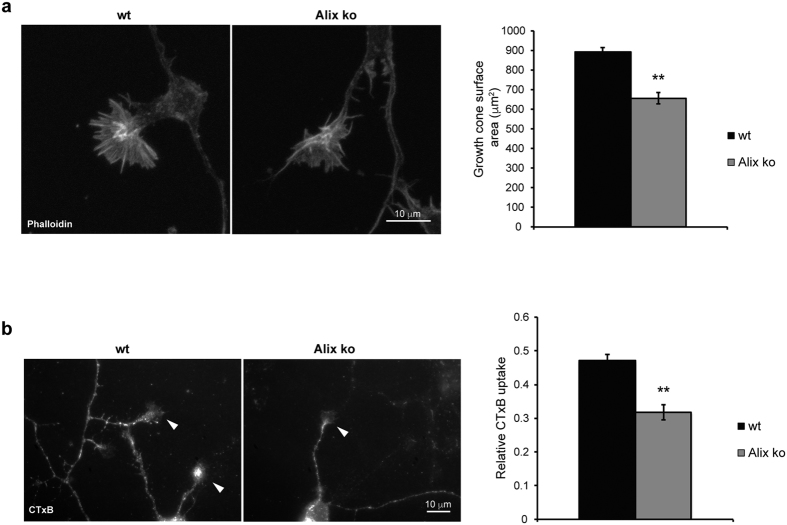
Functional impairment in growth cones of Alix ko neurons. (**a**) Growth cones of 1DIV neurons stained for F-actin with phalloïdin-Texas red. Quantification shows the reduction in the surface area of growth cones in Alix ko (n = 3 independent experiments **p < 0.01). (**b**) Relative CTxB endocytosis per growth cone is impaired in Alix ko neurons. Surface bound CTxB was removed and the fluorescence of internalized CTxB quantified. Arrowheads indicate growth cones where endocytosis was quantified (n = 3 independent experiments **p < 0.01).

Neither the size of the cell body nor the number of neurons in the radial dimension account for the reduced thickness of the adult Alix ko cortex (Supplementary Fig. S6a,b). Furthermore, the proportions of neurons on the total number of cells is the same in wt and ko adult cortices indicating that *alix* ablation does not modify the number of glial cells (Supplementary Fig. S6c). One major contributor to radial growth is post-natal dendritic outgrowth and we observed that dendritic arborisation of cortical pyramidal neurons is significantly reduced in Alix ko adult brains (Fig. 8a). This impairment seems intrinsic to Alix ko neurons since it was also observed in 2 week-old cultures of hippocampal neurons (Fig. 8b). Using the same neurons cultured for only 24 h, we demonstrated that *alix* ablation affects the early steps of neuritogenesis. Indeed, the number of neurites per neuron was significantly reduced (Fig. 8c). Furthermore neurite outgrowth was reduced and maturation of axons delayed (Fig. 8d,e). We correlated these impairments with a severe reduction in growth cone area, associated with an abnormal organization of actin filaments (Fig. 9a).

Growth cone dynamics are highly dependent on endocytosis which allows the fast recycling of surface molecules involved in growth cone movements^21^,^22^. Uptake of the B chain of Cholera toxin (CTxB), a prototype for cargoes entering cells through CIE^23^, was significantly reduced at growth cones of Alix ko neurons (Fig. 9b). Thus the requirement of Alix for CIE could explain why Alix ko neurons suffer from impaired dendrite arborisation resulting in the decrease of the post-natal radial expansion of cortical layers.

## Discussion

Most of the primary microcephalies such as that observed in Alix ko mice are due to defects in cell division during early embryonic cortical development^24^. *In vitro*, Alix downregulation was reported to lead to frequent cytokinesis defects dramatically increasing the number of cells with four and eight copy DNA content^25^,^26^,^27^,^28^. However, despite intensive study, we found no evidence that Alix ko neural progenitors suffer from cytokinetic defects which could have explained microcephaly. The absence of multi-nucleated somatic cells was also reported in *Drosophila alix* mutants even if completion of abscission during female germline stem cell division was selectively impaired^29^. The hypomorphic testes of Alix ko males could be related to the possible regulation of germ cell intercellular bridges by Alix^30^.

Alix ko microcephaly is partly explained by the loss of NPCs through apoptosis. The absence of apoptosis as in caspase-9 and -3 ko mice, leads to overgrowth of the neocortex^31^,^32^, while increase in apoptosis as a consequence of Notch or Ephrin deregulation, or of Brca1 (Breast cancer 1) deletion leads to microcephaly^33^,^34^,^35^,^36^. Our previous work has shown that Alix controls cell death by modulating caspase-9 and 8 activities^5^,^37^. Indeed, overexpression of Alix in the chick neural tube induces massive apoptosis of neuroepithelial cells leading to a 25% reduction in the width of the neural epithelium^4^. Thus, both the excess and the lack of Alix induce apoptosis of NPCs, suggesting that Alix expression level affects the balance of caspase activation regulators.

The radial unit hypothesis predicts that, if early enough, depletion of progenitors should affect expansion of the cortex mainly in its lateral but not its radial dimension except for deep layers containing the first born neurons^38^. Apoptosis of both RGCs and their neuron progenies in the Alix ko cortex suddenly rises around E11.5 and already returns to normal at E13.5. Noteworthy is that at E11.5, dying C3a + cells appear clustered in radial columns suggesting that apoptosis is activated in cells in close vicinity to dying RGC, possibly representing neurons migrating along clonally related radial glia^39^. Tbr2 + intermediate progenitors were never seen to be apoptotic, consistent with the observation that caspase-3 activity is returned to normal at E13.5, when the number of intermediate progenitors is massively increased. Therefore, the decrease in Tbr2 + progenitors seen at E13.5 correlates with the earlier demise of RGCs which give rise to these cells. The limited number of RGCs and neurons undergoing cell death during a very narrow time window allows the surviving RGCs to give rise to their progeny. This, together with the absence of neuron migration perturbation, explains why the cortex of Alix ko newborns appears almost normal apart from a reduction in its surface and in the thickness of layer VI, which is made by the first born neurons.

Apoptosis during development of the telencephalon is tightly regulated by cell-cell interactions, growth factors and interleukins like Notch, Wnt, EphA, FGF, which act in concert to control the fate of NPCs^18^,^33^,^40^,^41^. In Alix ko cultured NPCs, FGF signalization occurs faster and declines more slowly. As for many receptors, FGF-R activity is tightly regulated by endocytosis including clathrin-independent endocytosis CIE^15^,^16^. Hyper-activation of FGF signalization in the ko might thus result from the absence of regulation by Alix of CIE^3^ that we observed in NPCs. The abnormal FGF signalization seen in cultured NPCs correlates with their inability to develop into neurospheres when cultured in FGF-2. FGF signalization is required during early embryogenesis (E8.5-E9.5) for the survival of telencephalic precursor cells^42^, and later on (E12.5) to maintain the pool of radial glial cells by inhibiting their differentiation^14^,^18^,^19^,^42^,^43^. In mice in which FGF receptors were deleted during cortical neurogenesis, premature termination of neurogenesis impairing embryonic cortical expansion was detected by the increase in Tbr2 + cells^14^ whereas in Alix ko mice the number of Tbr2 + cells was decreased probably as a consequence of the death of their precursors. Thus, our results showing that Alix regulates FGF signaling reinforce the idea that fine tuning of this pathway is essential to control lateral expansion of the embryonic cortex. However, we do not exclude that impairments in clathrin-independent endocytosis due to the absence of Alix could affect other types of signalization and cell-cell interactions regulating cell survival in the embryonic telencephalon.

In Nestin;Alix^fl/fl^ mice, recombination occurs only in embryonic neural cells at a time when the absence of Alix is no longer deleterious^20^. In contrast to Alix ko, the Nestin;Alix^fl/fl^ adult cortex was only reduced in thickness but not in length (medio-lateral), an observation discriminating between two independent developmental time points at which Alix is required: during the early embryonic expansion of the cortex, when the absence of Alix compromises the survival of RGCs, and during the post-natal radial expansion of the cortical wall. Furthermore, since Nestin drives *alix* deletion only in neural cells this observation directly shows that the reduced thickening of the cortex is not an indirect effect of the compromised organization of the plexus choroid and consequent hydrocephaly recently described in another Alix ko mouse line^44^. The incapacity of the Alix ko cortex to expand radially after birth correlates well with the necessity of Alix expression in neurons for proper neurite outgrowth. A link between neurite outgrowth and brain expansion has already been reported in mice deleted of Rictor and PTEN, two antagonist regulators of the mTOR pathway influencing neurite outgrowth through microtubule and actin dynamics^45^. In Rictor ko mice, impairment in dendrite growth gives rise to small brains, while enhanced neurite growth might explain part of the macrocephaly phenotype of PTEN ko mice^12^,^46^. Growth and arborisation of neurites depend on growth cone dynamics driven by the actin network and endocytosis, which both appear abnormal in Alix ko neurons. The constant reorganization of the actin cytoskeleton is necessary to drive membrane protrusions, while endocytosis regulates surface expression of adhesion molecules and integrins needed to grow on different substrates^47^. The documented function of Alix both in the control of actin dynamics^44^,^48^,^49^ and endocytosis^3^ may explain how its absence induces growth cone alteration and defects in post-natal radial expansion of the cortical wall. Furthermore, the lack of Alix might also interfere with ESCRT regulation of dendrite pruning occurring directly^50^ or indirectly through the endosomal system^51^.

Human microcephalies affect 2% of the general population and have complex molecular and cellular bases remaining poorly understood. In primary microcephalies also called microcephaly primary hereditary (MCPH), the brain and cortex organization remain grossly normal^52^. A number of ko mouse lines have been made to model MCPH. When not lethal, they lead to microcephalies accompanied by defects in brain architecture^24^. Alix ko microcephaly is to our knowledge unique in that it affects only the lateral expansion of the embryonic cortex and its radial expansion after birth and may therefore prove to be an invaluable model for understanding some of the mechanisms underlying brain growth.

## Material and Methods

### Generation of Alix knock out mice

C57BL/6 N mice with an *alix* floxed allele were generated by la Clinique de la souris (Strasbourg, France). Briefly, a flippase recognition target (FRT)‐flanked neomycin cassette was inserted downstream of exon 2 of the *alix* gene (chromosome 3). The *loxP* sites were inserted upstream and downstream of exon 2. The floxed mice were then backcrossed with Rosa26-FlpE mice to remove the neomycin cassette and with either Actin-cre or Nestin-cre mice^53^,^54^ (The Jackson laboratory) to obtain complete ko (Alix ko) or conditional ko restricted to the brain (Nestin;Alix^fl/fl^) respectively. In both cases, the Cre recombination changes the reading frame introducing a stop codon at 10718 bp of *alix* gene thus abolishing the expression of Alix protein.

### Animals

In accordance with the policy of the Institut des Neurosciences de Grenoble (GIN) and the French legislation, experiments were done in compliance with the European Community Council Directive of 24 November, 1986 (86/609/EEC). The research involving animals was authorized by the Direction Départementale de la protection des populations—Préfecture de l’Isère-France (Fraboulet S., PhD, permit number 381002) and by the ethics committee of GIN n 004 accredited by the French Ministry of Research.

Pregnant females were sacrificed, and brains isolated from embryos. Adult mice of both sexes were sacrificed by cervical dislocation to obtain organs. Newborn pups (P0) were used for primary neuron cultures.

### Antibodies and other reagents

See Table 1 for antibodies used.

Reagents used: Cresyl violet, haematoxylin, eosin, BrdU, laminin, poly-D-lysin, phalloidin-Texas red, Hoechst 33258 (Sigma), TUNEL *In Situ* Cell Death Detection kit, protease inhibitors EDTA-free (Roche), FD Rapid Golgistain kit (generous gift of Dr F. Du, FD Neurotechnologies), CTxB (Molecular probes), lipofectamine 3000 (Thermofischer scientific), Vectastain Elite ABC HRP kit (Vector), recombinant human EGF and FGF-2 (PeproTech).

### Cell culture and transfections

Primary cultures of neural progenitor cells (NPCs) were prepared as follow: cortices from E12.5 embryos were dissected in Hanks’ balanced salt solution (HBSS, 2 mM MgSO_4._7H_2_0, 10 mM HEPES, 0.1% penicillin/streptomycin). Dorsal telencephalons were isolated by removing ganglionic eminences and treated with 0.05% trypsin- 0.53 mM EDTA for 10 min at 37 °C. Cells were then mechanically dissociated and seeded at 20,000 cells/cm2 in DMEM/F12 medium (2 mM glutamine, 1% B27, 20 ng/ml EGF and 10 ng/ml FGF-2) on coverslips coated with 100 μg/ml poly-D-lysine and 20 μg/ml laminin. For neurospheres, 1000 cells were plated in uncoated 96-well plates in DMEM/F12 medium supplemented with 2 mM glutamine, 1% B27 and either FGF-2 alone (10 ng/ml) or together with EGF (20 ng/ml). After 6 days in culture, the total number of neurospheres per well was counted (3 wells per embryo).

Primary cultures of hippocampal neurons were prepared and cultured as previously described^55^. Neurons were transfected at 10 days *in vitro* (DIV) using a calcium phosphate method optimized for mature neuronal cultures as previously described^55^.

### Histology

Heads of embryos (E11.5-13.5) and brains of newborn (P0) were dissected in PBS and fixed from 1.5 h to overnight at 4 °C with 4% paraformaldehyde-4% sucrose (PFA-sucrose) in 120 mM phosphate buffer (PB) pH 7.4. For cryosectioning, the samples were equilibrated at 4 °C in 30% sucrose in PB and then embedded in 15% gelatin in 30% sucrose-PB and frozen. Cryostat sections were cut at 14 μm for embryos and 16 μm for P0.

Adult brains were dissected and either frozen in chilled isopentane for cryosectioning or fixed in Carnoy’s solution (60% ethanol, 30% chloroform, 10% acetic acid) for 4 h before paraffin embedding and sectioning at 7 μm (16 μm for cryosections). Sections were stained with haematoxylin-eosin or Cresyl violet.

Surface areas of sections of various brain structures, including cortex, striatum, hippocampus, thalamus and cerebellum, were quantified by imageJ on photographs of 16 μm cryosections, stained with Cresyl violet, taken at equivalent levels. We used sections at Bregma 0.98 mm for whole brain, cortex and striatum, Bregma −1.46 mm for hippocampus and hypothalamus and Bregma −5.80 mm for cerebellum.

The thickness and medio-lateral lengths of the cortex were also measured on 7 μm paraffin sections stained with haematoxylin-eosin, at Bregma 0.98 mm for adults and the corresponding level for P0. We used the outer limits of the brain on each section to measure the medio-lateral length and a line parallel to the inter-hemispheric furrow in the motor cortex M2 for the measurement of the thickness.

Testes were fixed overnight in Bouin’s fluid before paraffin embedding, sectioning and staining with haematoxylin-eosin.

### Immunofluorescence and immunohistochemistry

Cryosections were permeabilized in 0.3% Triton X-100 prior immunofluorescent stainings in 0.3% Triton X-100, 10% Goat Preimmune serum (GPi). Antigen retrieval was performed for the antibodies indicated (See Table 1) by boiling in a cooker in 10 mM sodium citrate pH6, 0.1% Tween20.

For paraffin sections, NeuN was revealed by immunohistochemistry as described previously^56^.

For cell cultures, NPCs or neurons were fixed by 4% PFA (or PFA-sucrose for neurons) and immunostained after permeabilization (see Table 1).

### BrdU labeling

BrdU (100 mg/kg) was injected intraperitoneally into pregnant females at 12.5 days *post-coitum*. Mice were sacrificed 1.5 h after injection, and embryos fixed and sectioned as described above. For BrdU immunofluorescence, cryosections were subjected to 2 M HCl at 37 °C and washed in borate buffer 100 mM, pH 8.5 before immunostaining.

### TUNEL labeling

To visualize DNA fragmentation, the terminal deoxynucleotidyl transferase-mediated dUTP-biotin nick end labeling (TUNEL) method was used^57^.

### Golgi staining

For Golgi staining impregnation, we used FD Rapid GolgiStain kit. Whole brains were immersed for 4 days and treated as described by the manufacturer. In each of three animals per genotype, cortical neurons from layers V/VI were submitted to Sholl analysis. Starting from the soma, they were traced by hand following dendritic and axonal processes on a thickness of 100 μm and Sholl analysis was performed.

### Analysis of cell cycle by FACs

Dissociated NPC were fixed in 70% ethanol at 4 °C overnight. Then they were centrifuged (500 g 10 min) and resuspended in PBS containing 50 μg/ml propidium iodide, 100 μg/ml Rnase A and analysed by flow cytometry (BD Accuri C6).

### FGF-2 signaling

After 3 DIV, NPCs were starved for 2 h in DMEM/F12 medium containing 2 mM glutamine, 1% B27, then incubated with 10 ng/ml FGF-2 for 5, 10 or 30 min. Cells were immunostained for the total and the phosphorylated forms of ERK1/2. The ratio of P-ERK/ERK intensity was calculated and normalized to values at t = 0 min, considered as 1.

### GPI-GFP internalization

NPCs were transfected at 3 DIV by GPI‐GFP (gift G. Van der Good, EPFL, Switzerland) using lipofectamine 3000. At 6 DIV, cells were incubated for 20 min on ice with an anti‐GFP after rinsing in cold DMEM/F12 containing 0.2% BSA and 20 mM HEPES. Cells were then either washed in HBSS and fixed or incubated at 37 °C for 5 min in DMEM/F12 containing 0.2% BSA to allow endocytosis. Non-endocytosed GPI-GFP was stripped (200 mM acetic acid, 500 mM NaCl, pH 2.4). Internalized antibody was detected by immunostaining after fixation and permeabilization. For quantification, internalized fluorescence at 37 °C was normalized to the fluorescence at 4 °C.

### CTxB internalization

Neurons (2 DIV) were washed with cold Neurobasal medium containing 0.2% BSA and 20 mM Hepes, and incubated with 2 μg/ml CTxB-TRITC for 5 min at 4 °C. Neurons were washed in HBSS and either fixed in PFA-sucrose or incubated for 2 min at 37 °C in Neurobasal containing 0.2% BSA to allow internalization. Surface bound CTxB was stripped as above and neurons fixed. For uptake quantification, growth cones were labeled with an anti-Tau antibody, and the mean CTxB-TRITC fluorescence intensity was measured inside the growth cone after background subtraction. Intensities were normalized to binding at 4 °C.

### Fluorescent microscopy

Images were acquired by Metamorph with a Zeiss Axiovert microscope using 5x, 10x, 20x, 40x objectives or 63x Plan-Apochromatic oil immersion objective (NA 1.3, Zeiss) or by Zen software with a Zeiss LSM 710 inverted confocal microscope with 20x, 40x oil objective (NA 1.3, Zeiss) or 63x oil objective (NA 1.4, Zeiss). Laser lines at 488 nm, 561 nm and 633 nm were used for exciting fluorophores. Pinhole size was usually set to generate 1 μm-thick optical slice.

### Magnetic Resonance Imaging

MRI was performed at the IRMaGE platform (GIN, France) on the 7 T Bruker Biospec Avance III using a volume transmit/surface receive coil combination. The anaesthetized animal (1.5% isoflurane in air) was placed in a heated cradle, regulated at 37 °C. T2-weighted spin-echo images (TR/TE = 5,000/42 ms, field of view = 15 × 15 mm^2^, coronal slice thickness = 0.6 mm, 30 slices) were obtained across the entire brain. Subsequently, the contours of ventricle and brain were manually delineated on the T2-weighted images.

### Electron microscopy

The antero-dorsal part of the developing cortex was dissected from E12.5 embryos and fixed for 2 h in 2.5% glutaraldehyde, 2% PFA in 0.1 M PB, pH 7.3, post-fixed in 1% osmium tetroxide and embedded in Epon. Sections were stained classically and observed with a 1200EX JEOL TEM microscope.

### Tissue preparation for Western blotting

Mouse organs were homogenized in ice-cold isotonic lysis buffer (0.32 M sucrose, 4 mM HEPES, pH 7.4, protease and phosphatase inhibitors). The homogenate was centrifuged at 1400 g for 10 min. The postnuclear supernatant was further homogenized by adding 0.5% Triton-X100 to the lysis buffer, stirred 30 min at 4 °C and cleared at 12 000 g for 10 min. Tissue lysates were resuspended in Laemmli buffer and processed for Western blotting as described previously^3^,^55^.

### Statistical Analyses

Results shown are means, and error bars represent standard errors of the mean (s.e.m). Experiments were repeated at least 3 times. Statistical significance was determined either by two-tailed Student’s t-test (2 groups) or by one-way Anova and Dunnett’s test (more than 2 groups) as appropriate. For multiple comparisons of Mendelian inheritance and the number of primary neurites, statistical significance was determined by Pearson’s Chi-square test. For FGF-2 signaling, curves were fitted with 2^nd^ degree polynomial regression function (quadratic fit) and statistical significance was determined by one-way Anova and Dunnett’s test.

In all experiments, statistical significance is indicated as follows: *p < 0.05; **p < 0.01; ***p < 0.001.

## Additional Information

**How to cite this article**: Laporte, M. H. *et al*. Alix is required during development for normal growth of the mouse brain. *Sci. Rep.* **7**, 44767; doi: 10.1038/srep44767 (2017).

**Publisher's note:** Springer Nature remains neutral with regard to jurisdictional claims in published maps and institutional affiliations.

## Supplementary Material

Supplementary Information

## Figures and Tables

**Table 1:** Antibodies used in this study.

Antibody	Species	Compagny (Reference)	Dilution		
			IHC	IF	WB
Anti-Actin	Mouse	Millipore (MAB1501R)			10,000
Anti-Alix	Rabbit	Covalab (ab0204)	100*		10,000
Anti-βcatenin	Mouse	Sigma (C7082)	500		
Anti-BrdU	Rat	Eurobio-abCys S.A. (ABC117-7513)	1000		
Anti-cleaved caspase3	Rabbit	Cell signaling (551150)	700	1000	2000
Anti-cTip2	Rat	Gift from P. Gressens laboratory	500*		
Anti-GFP	Rabbit	Life Technologies (A11122)		500	
Anti-Iba1	Rabbit	Eurobio-abCys S.A. (CP290B)	200		
Anti-MAP2	Mouse	Chemicon (AB 5622)		500	
Anti-RC2	Mouse	DSHB (AB_531887)	10		
Anti-NeuN	Mouse	Gift from A. Buisson’s laboratory	250		
Anti-p44/42 MAPK	Rabbit	Cell signaling (4695)		1000	
Anti-phospho-p44/42 MAPK (T202/Y204)	Mouse	Cell signaling (9106)		1000	
Anti-phopho-Histone H3 (S10)	Rabbit	Millipore (06–570)	500		
Anti-Tau	Rabbit	Dako (A002401)		5000	
Anti-Tbr2	Rabbit	Abcam (ab23345)	200*		
Anti-βIIItubulin (Tuj1)	Mouse	Covance (MMS-435P)	500		
Anti-Mouse biotinylated	Goat	Vector, Burlinghame, USA (BA-9200)	500		
Anti-Mouse HRP	Goat	Jackson (115-035-166)			10,000
Anti-Rabbit HRP	Goat	Jackson (115-035-044)			10,000
Anti-Mouse A488	Goat	Molecular Probes (A-11029)	500	1000	
Anti-Mouse A594	Goat	Molecular Probes (A-11037)	500	1000	
Anti-Rabbit A488	Goat	Molecular Probes (A-11034)	500	1000	
Anti-Rabbit A594	Goat	Molecular Probes (A-11037)	500	1000	
Anti-Rat A488	Goat	Molecular Probes (A-11006)	500	1000	

The antibody dilutions we used are indicated for each application. IHC, immunohistochemistry; IF, immunofluorescence; WB, Western blotting. *Antigen retrieval.
