# Supplementary material for: Alix is required during development for normal growth of the mouse brain
Source: Sci Rep. 2017 Mar 21;7:44767. doi: 10.1038/srep44767 (PMC5359572; doi:10.1038/srep44767)
Supplement: Supplementary Information [file srep44767-s1.pdf]

Alix is required during development for normal growth of the mouse brain.

Marine H. Laporte[1,2], Christine Chatellard[1,2], Victoria Vauchez[1,2], Fiona J. Hemming[1,2], Jean-Christophe Deloulme[1,2], Frédérique Vossier[1,2], Béatrice Blot[1,2], Sandrine Fraboulet[1,2*] and Rémy Sadoul[1,2,*]

1) Institut National de la Santé et de la Recherche Médicale (INSERM), U1216, F-38042 Grenoble, France

2) Université Grenoble Alpes, Institut des Neurosciences, F-38042 Grenoble, France

* Corresponding authors

email: remy.sadoul@univ-grenoble-alpes.fr; sandrine.fraboulet@univ-grenoble-alpes.fr

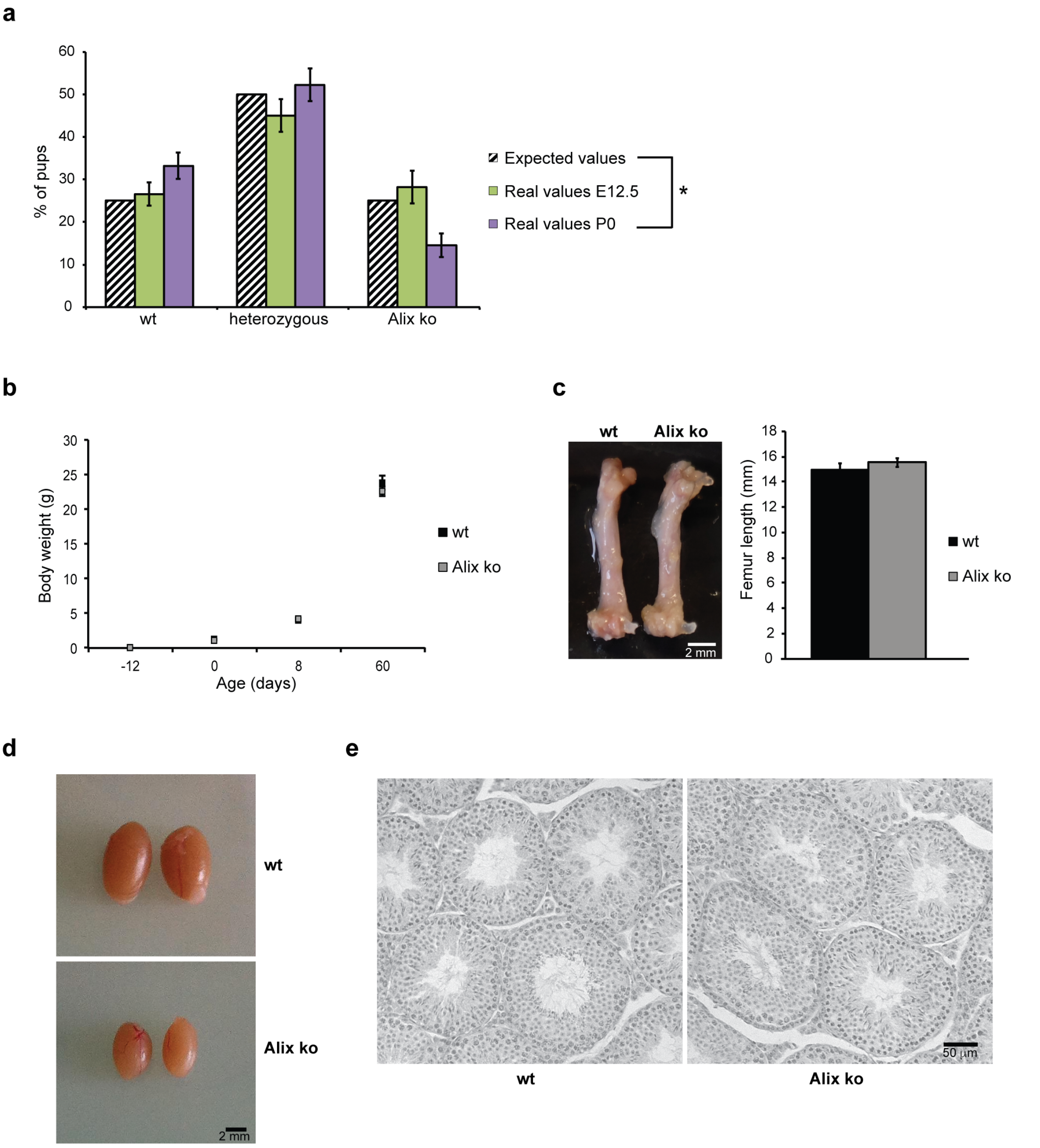

Supplementary Figure 1. Physical features of Alix ko mice. (**a**) Mendelian inheritance in Alix heterozygous (Hz) progeny is abnormal: fewer Alix ko pups than expected were born from crossing Hz mice. However, the expected number of Alix ko E12.5 embryos was found (n=20 animals). (**b**) Weight gain from E12 to adult and (**c**) size of adults are equivalent in wt and Alix ko (n=6 animal for each age). (**d,e**) Alix ko testes are dramatically reduced (**d**) with normal organization of seminiferous tubules (**e**).

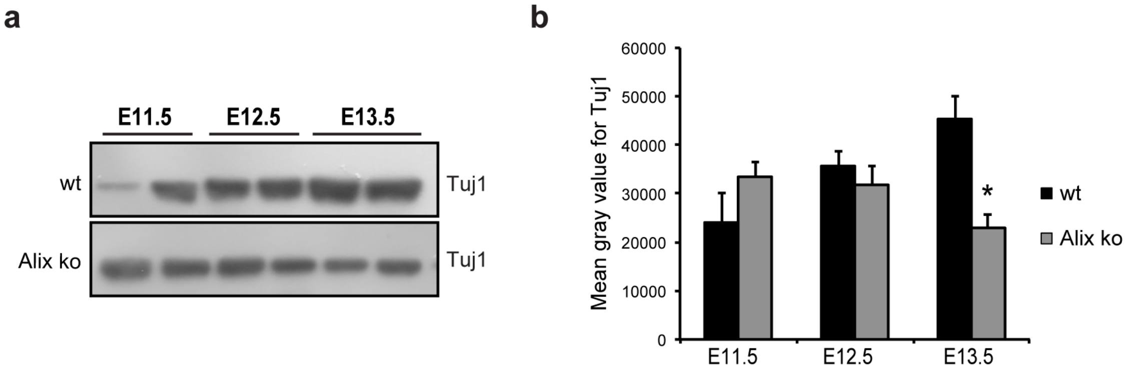

Supplementary Figure 2. Western blot demonstrating the reduction of Tuj1 protein only in E13.5 Alix ko cortices compared to wt cortices (n=2 animals *p<0.05).

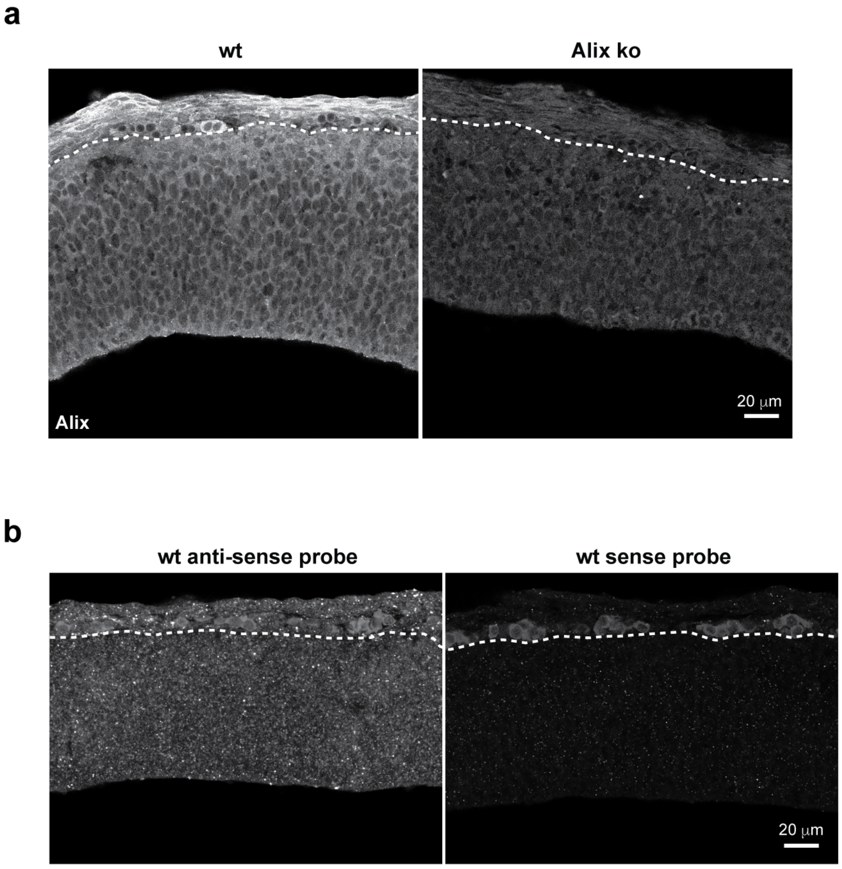

Supplementary Figure 3. **Alix is homogeneously expressed in the cortex.** Alix protein, revealed by immunofluorescence (**a**) and mRNA, detected by *in situ* hybridization (**b**), are homogeneously distributed in the cortical wall of wt E12.5 embryos. Dashed lines delimit the basal surface of the cortex.

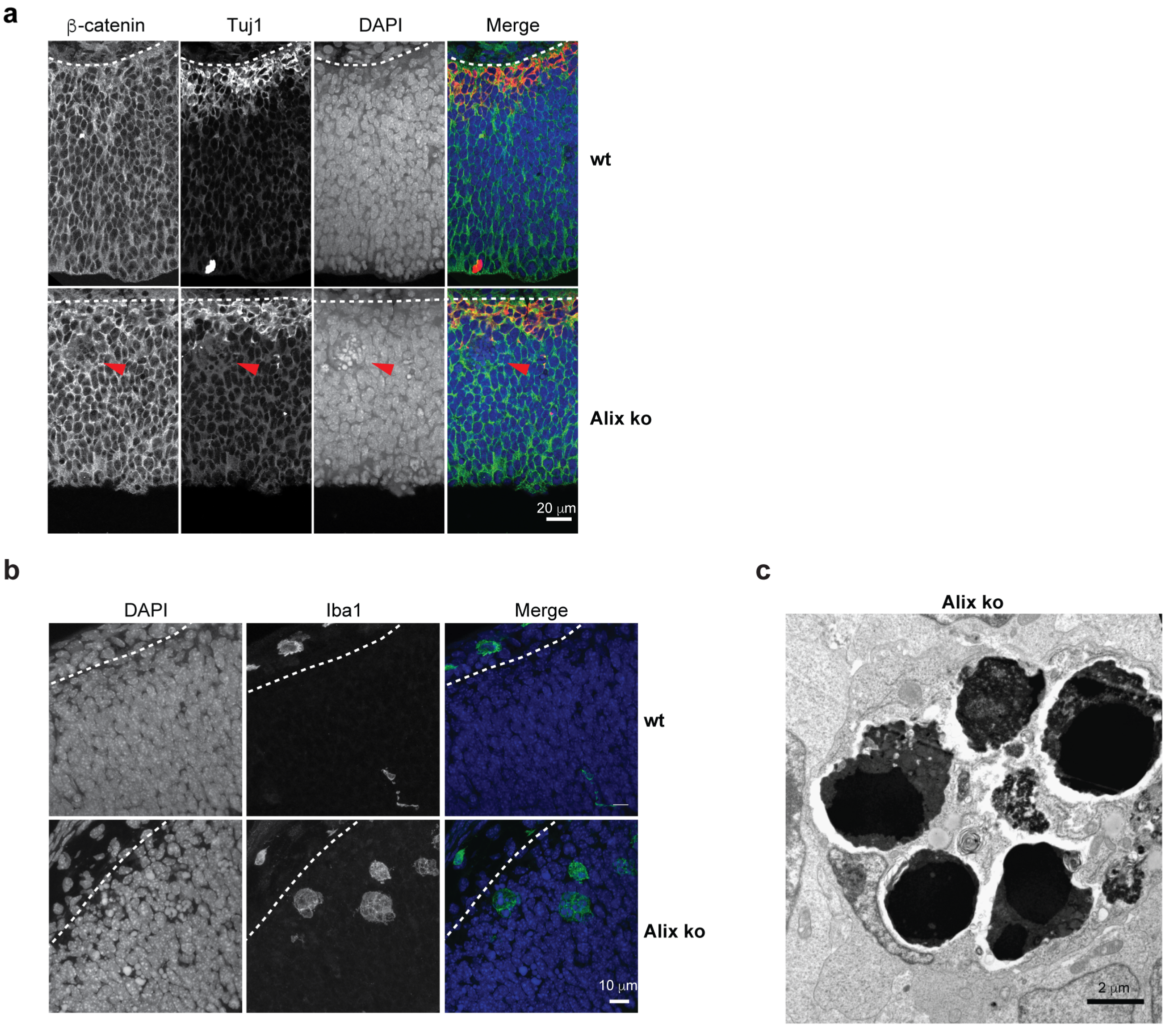

Supplementary Figure 4. Clusters of dying cells are engulfed by Iba1+ microglia at E12.5.

(**a**) Coronal section labelled with anti-β-catenin, anti-Tuj1 antibodies and DAPI showing an aggregate of pyknotic nuclei (red arrowheads). Dashed lines delimit the basal surface of the cortex. (**b**) Anti-Iba1 staining shows microglial cells engulfing pyknotic nuclei throughout the Alix ko telencephalon whereas none is seen in wt cortices. Dashed lines delimit the basal surface of the cortex. (**c**) Clump of apoptotic cells surrounded by an engulfing, putative microglial cell observed by EM in sections of Alix ko cortices.

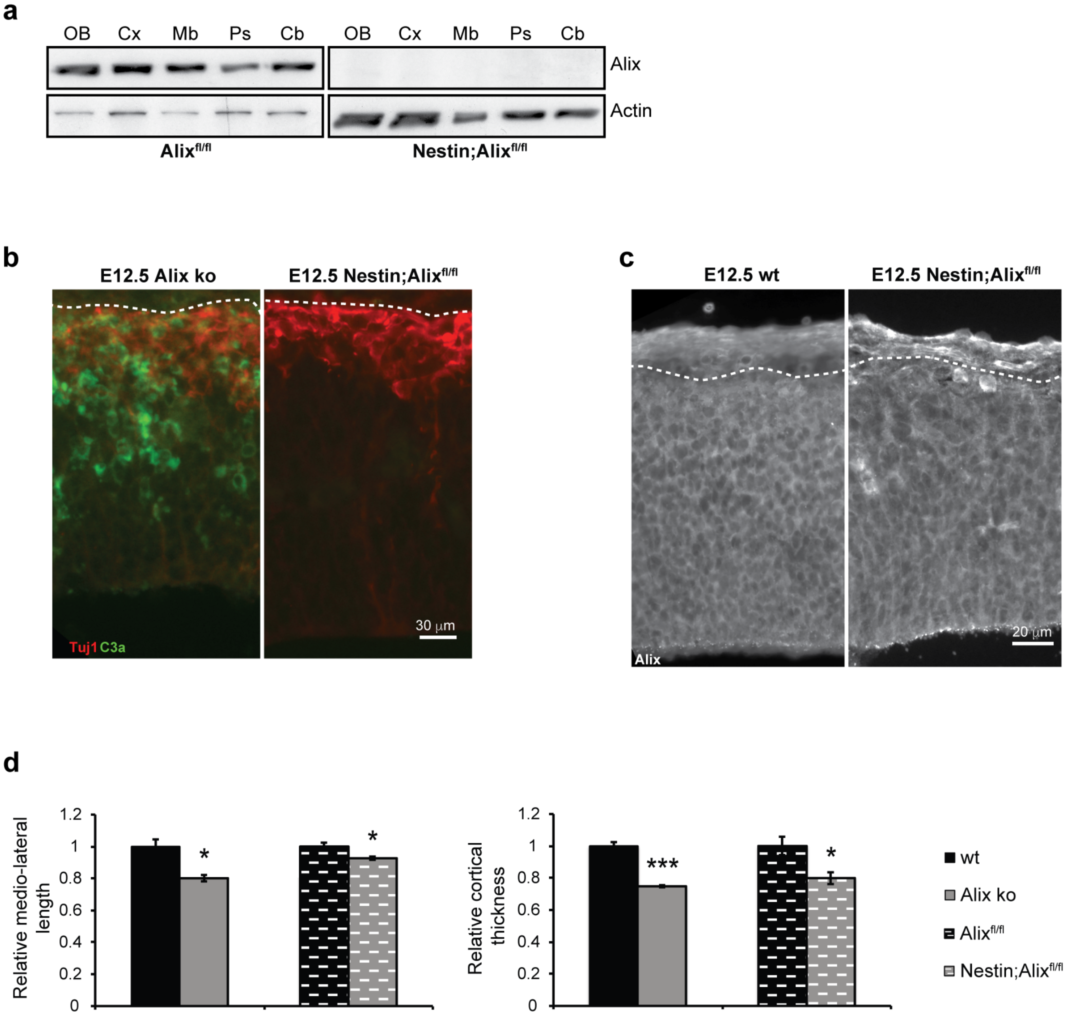

Supplementary Figure 5. Conditional ablation of Alix in Nestin;Alix$^{fl/fl}$ mice abolishes caspase-3 activation in embryos and mainly affects the thickness. (**a**) Western blot demonstrating the lack of Alix expression in olfactory bulb (OB), cortex (Cx), midbrain (Mb), pons (Ps) and cerebellum (Cb) of adult Nestin;Alix$^{fl/fl}$ mice . (**b**) C3a+ cells are undetectable in sections of E12.5 of Nestin;Alix$^{fl/fl}$ cortex immunostained with anti-Tuj1 (red) and anti-C3a (green). For comparison the left photograph (wide field) shows an equivalent section of an Alix ko embryo. (**c**) Alix protein, revealed by immunofluorescence is homogeneously distributed in the cortical wall of wt and Nestin;Alix$^{fl/fl}$ embryos. Dashed lines delimit the basal surface of the cortex.

(**d**) Relative medio-lateral length (left) and cortical thickness (right) of adult cortex. The difference in cortical thickness between Nestin;Alix$^{fl/fl}$ and Alix$^{fl/fl}$ is identical to that seen between Alix ko and wt, whereas the difference in medio-lateral size is far less pronounced (n=4 animals).

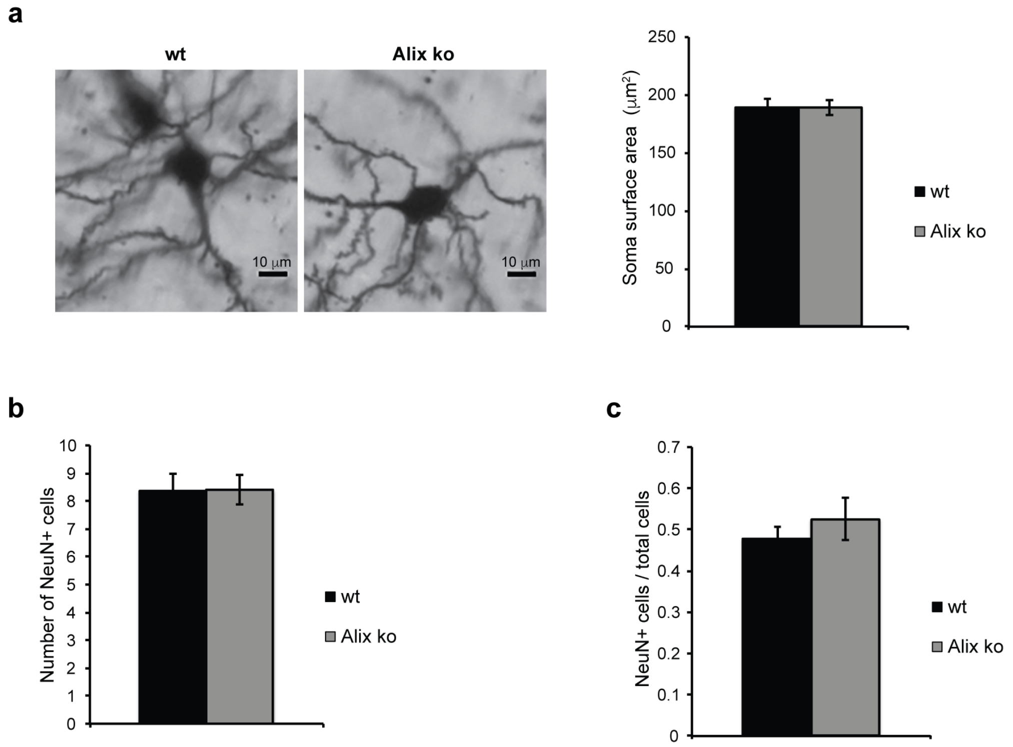

Supplementary Figure 6. The cell number and soma volume of cortical neurons is not different between wt and Alix ko animals. (**a**) Golgi stained neurons in layers V-VI of the adult cortex. The size of neuronal cell bodies does not vary between Alix ko and wt mice (n=3 animals). (**b**) The number of NeuN+ neurons, counted along a radial line in the adult cortical wall, is not affected by the lack of Alix (n=4 animals). (**c**) The ratio of the number of NeuN+ neurons to the total number of cells stained with haematoxylin-eosin, does not differ between Alix ko and wt cortices. (n=4 animals).

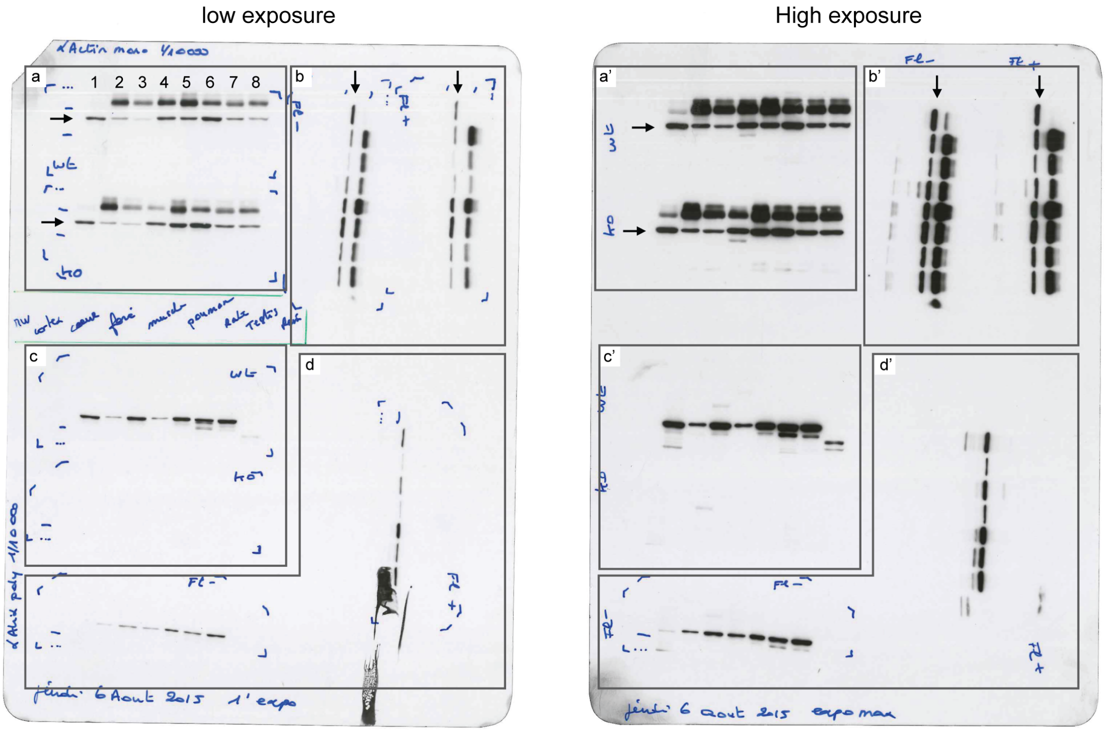

Supplementary Figure 7. Full length western blot showing Alix expression in organs of wt, Alix ko, Alix$^{fl/fl}$ and Nestin;Alix$^{fl/fl}$ animals, shown as cropped images in Figure 1. (**a,a'**) Actin expression in different organs of wt, Alix ko animals from low (**a**) and high (**a'**) film exposures (arrows represent specific band for Actin). (**b,b'**) Actin expression in different organs of Alix$^{fl/fl}$ and Nestin;Alix$^{fl/fl}$ animals from low (**b**) and high (**b'**) film exposures (arrows represent specific band for Actin). (**c,c'**) Alix expression in different organs of wt, Alix ko animals from low (**c**) and high (**c'**) film exposure showing the absence of Alix expression in Alix ko organs. (**d,d'**) Alix expression in different organs of Alix$^{fl/fl}$ and Nestin;Alix$^{fl/fl}$ animals from low (**d**) and high (**d'**) film exposures showing the strong reduction of Alix expression in Nestin;Alix$^{fl/fl}$ brain.

1: brain; 2: heart; 3: liver; 4: muscle; 5: lung; 6: spleen; 7: testis; 8: kidney (note the short form of Alix presents in the kidney).

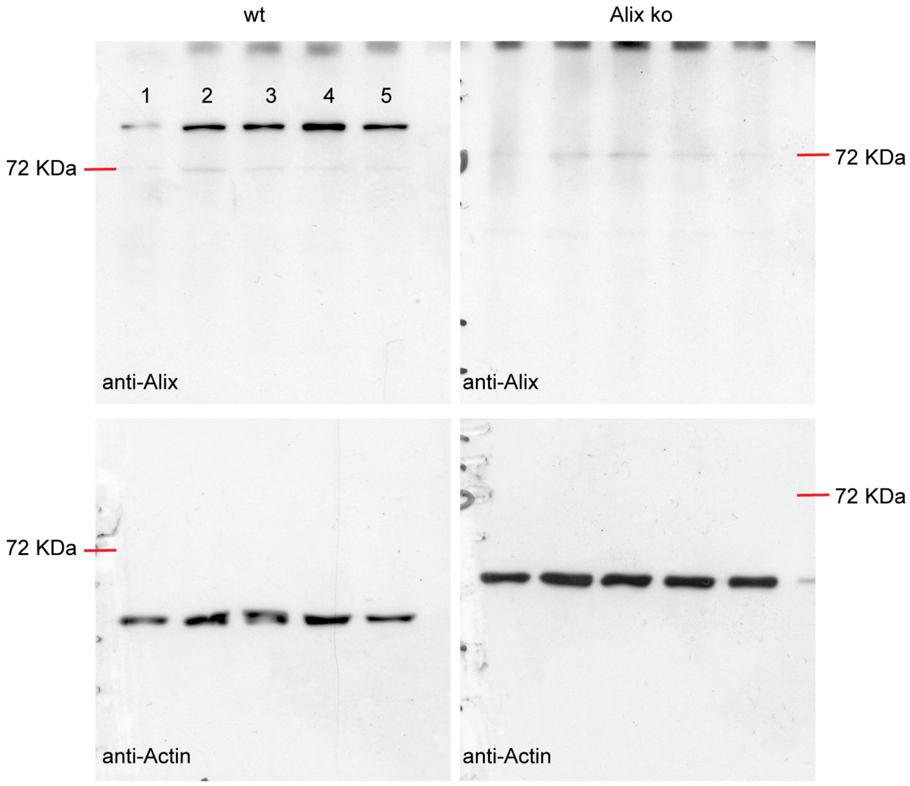

Supplementary Figure 8. Full-length western blot showing Alix expression in brain regions of wt and Alix ko, shown as cropped images in Figure 2. Alix (upper panel) and Actin (lower panel) expression in different brain regions of wt (left) and Alix ko (right) showing the strong reduction of Alix expression in all brain regions of Alix ko examined.

1: olfactory bulb; 2: cortex; 3: midbrain; 4: pons; 5: cerebellum.

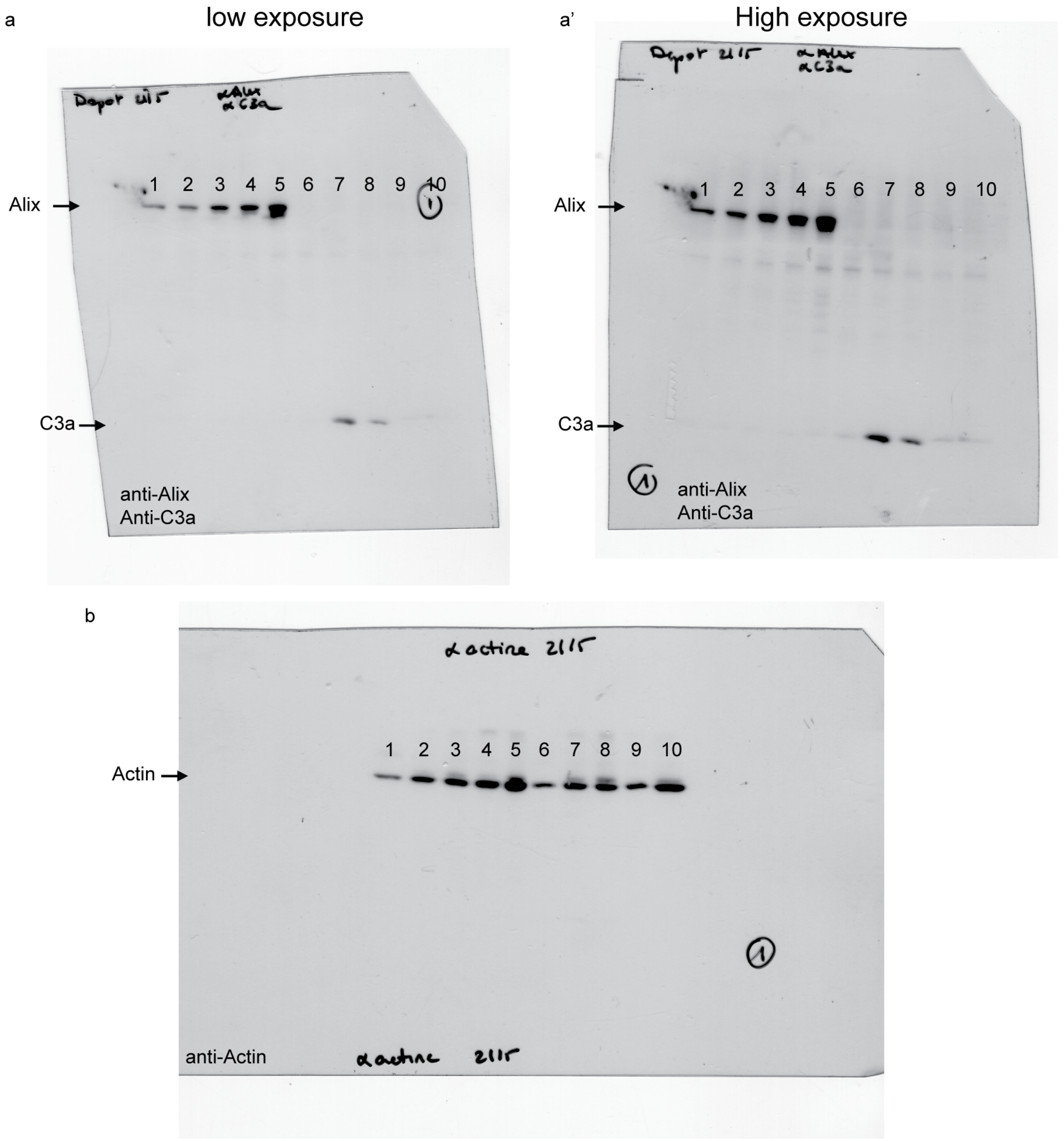

Supplementary Figure 9. Full-length western blot of extracts from dorsal telencephalons shows C3a detection in E11.5 and E12.5 Alix ko cortices, shown as cropped images in Figure 6. (**a**,**a'**) Alix and C3a expression in extracts from dorsal telencephalon of wt and Alix ko embryos from low (**a**) and high (**a'**) film exposure. (**b**) Actin expression in extracts from dorsal telencephalon of wt and Alix ko embryos.

1: wt E10.5; 2: wt E11.5; 3: wt E12.5; 4: wt E13.5; 5: wt E15.5; 6: Alix ko E10.5; 7: Alix ko E11.5; 8: Alix ko E12.5; 9: Alix ko E13.5; 10: Alix ko E15.5.
